# Successful IMRT and concurrent chemotherapy for a patient with intrathoracic extensive‐stage small cell lung cancer

**DOI:** 10.1002/rcr2.919

**Published:** 2022-03-09

**Authors:** Yoshitsugu Horio, Hiroyuki Tachibana, Junichi Shimizu, Waki Hosoda, Yutaka Fujiwara

**Affiliations:** ^1^ Department of Outpatient Services Aichi Cancer Center Hospital Nagoya Japan; ^2^ Department of Thoracic Oncology Aichi Cancer Center Hospital Nagoya Japan; ^3^ Department of Radiation Oncology Aichi Cancer Center Hospital Nagoya Japan; ^4^ Department of Pathology and Molecular Diagnostics Aichi Cancer Center Hospital Nagoya Japan

**Keywords:** chemoradiotherapy, IMRT, intrathoracic nonregional LN metastasis, small cell lung cancer

## Abstract

Treatment of extensive‐stage (ES) small cell lung cancer (SCLC) is a challenge with poor local control and dismal overall survival. Although single extrathoracic metastasis was defined as M1b according to the eighth edition of the tumour–node–metastasis (TNM) classification of lung cancer, M1b includes involvement of a single intrathoracic nonregional lymph node (LN) such as pericardial, internal mammary or paravertebral LNs. Here, we report a successful treated case of a 50‐year‐old female with ES‐SCLC with right pericardial LN involvement, cT1cN3M1b (LYM). She initially received two cycles of induction chemotherapy consisting of cis‐Diamminedichloroplatinum/cisplatin (CDDP) and etoposide and achieved a very good partial response. She then received curative chemoradiotherapy with intensity‐modulated techniques (45 Gy in 30 fractions BID), followed by an additional cycle of chemotherapy. She is free of recurrence for more than 2.5 years.

## Graphical abstract

This is the first reported case of intrathoracic extensive‐stage small cell lung cancer, successfully treated with intensity‐modulated radiation therapy and concurrent chemotherapy.

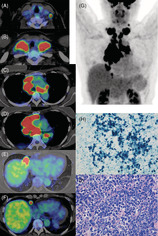

## INTRODUCTION

Small cell lung cancer (SCLC) is the most common and aggressive pulmonary neuroendocrine carcinoma, accounting for about 15% of all diagnosed lung cancer cases.^1^ Since the late 1950s, the Veterans Administration Lung Cancer Study Group (VALSG) staging system divided SCLC into limited‐stage (LS) or extensive‐stage (ES) SCLC.^2^ LS‐SCLC was initially characterized as tumoural involvement limited to one hemithorax (with or without local extension) with no distant extrathoracic metastatic disease and inclusion in a single radiation port, and then a modified version of VALSG staging for SCLC included ipsilateral supraclavicular lymph nodes (LNs) and contralateral mediastinal or supraclavicular LNs and ipsilateral pleural effusions. However, the International Association for the Study of Lung Cancer (IASLC) recommended to use the seventh edition of the American Joint Committee on Cancer (AJCC) tumour–node–metastasis (TNM) staging system for lung cancer instead of the VALSG staging system.^3^ The recent criteria of the eighth edition of the TNM staging system also correspond to stages I–III and stage IV for LS‐SCLC and ES‐SCLC, respectively.^4^ Although most clinicians and clinical trials blend the modified VALSG and IASLC criteria by classifying contralateral mediastinal and ipsilateral supraclavicular LN involvement as LS‐SCLC, tumoural involvement with intrathoracic LNs beyond the nodal stations shown in the IASLC LN map of lung cancer, such as internal mammary, peri(para)cardiac and paravertebral LNs was classified as ES‐SCLC.

Although patients with SCLC have been treated with systemic chemotherapy with or without radiation therapy (RT) and a significant minority of patients with SCLC are amenable to surgical resection, immune checkpoint inhibitors have been recently incorporated into the treatment for ES‐SCLC.^1^ Consolidative thoracic RT (TRT) is beneficial for selected patients with ES‐SCLC with complete response or good response to systemic therapy, especially with residual thoracic disease and low‐bulk extrathoracic metastatic disease.^5^, ^6^

Intensity‐modulated RT (IMRT) is an innovative radiation technique that optimizes the dose distribution in three dimensions (3D) by focusing radiation on tumour burdens from multiple directions with nonuniform dose intensity in the radiation field, thereby reducing the dose to normal tissues around the tumour and surrounding organs.^7^ It has a very wide range of applications, including large tumours or targets that are difficult to treat with ordinary 3D‐RT. NRG/RTOG 0617 trial showed that patients treated with IMRT had significantly less G3–5 pneumonitis and lower heart doses in locally advanced non‐SCLC. However, limited data on IMRT are available in SCLC. Here, we report a case of a patient of ES‐SCLC with a right pericardial LN involvement who was successfully treated with chemoradiotherapy and remained recurrence‐free for more than 2.5 years.

## CASE REPORT

**FIGURE 1:**
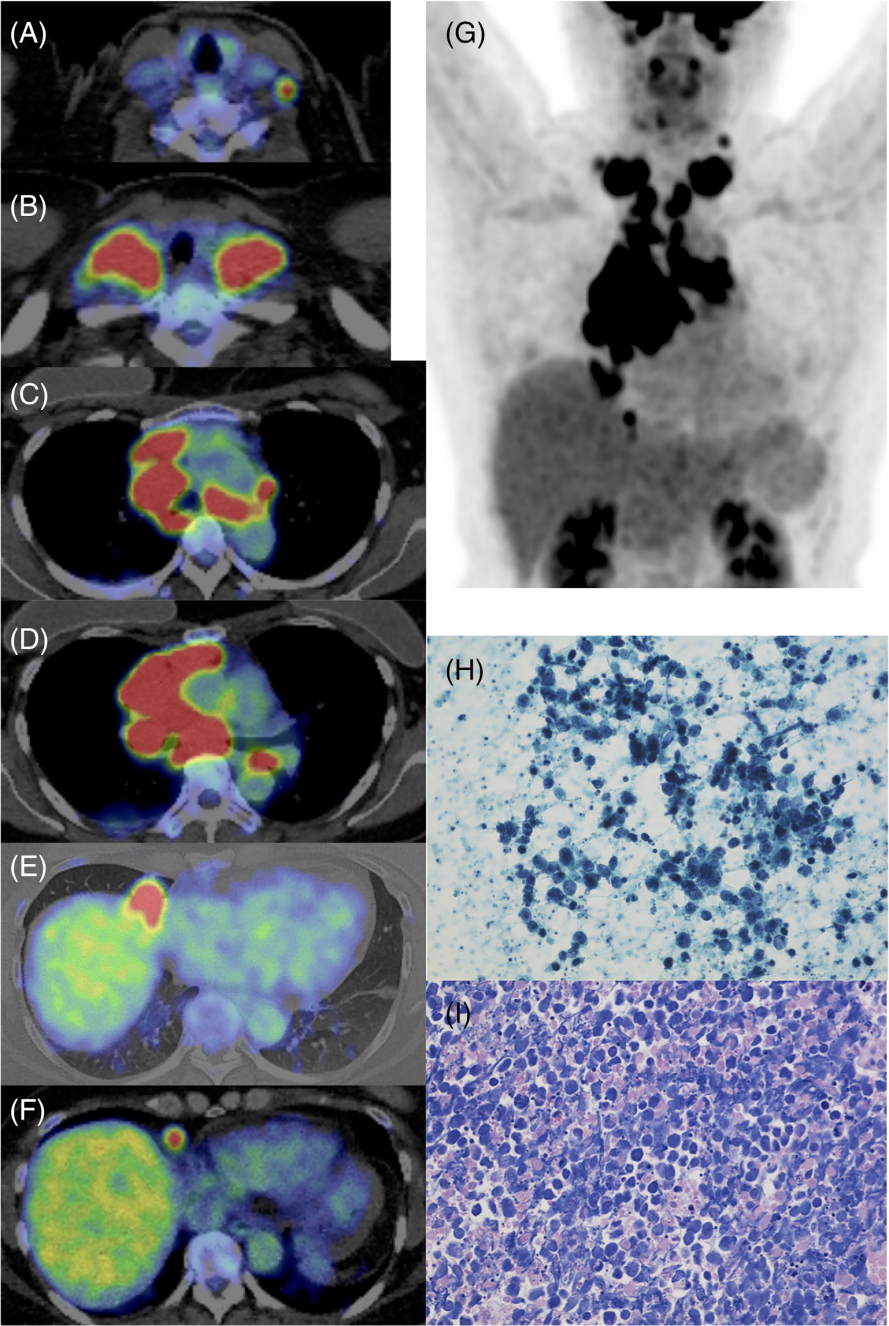
A 50‐year‐old woman underwent positron emission tomography‐computed tomography (PET‐CT) scan and aspiration cytology examination and was diagnosed with intrathoracic extensive‐stage small cell lung cancer (SCLC). Axial fused PET‐CT images (A–F) and anterior view of the maximum intensity projection (MIP) image of the PET‐CT scan (G) revealed increased [18F] fluorodeoxyglucose uptake of the bilateral supraclavicular, bilateral mediastinal and right pericardiac lymph nodes (LNs) and the right middle lobe tumour and a small amount of right pleural effusion. Aspiration cytology (H) and cell block preparation (I) of the left supraclavicular LN demonstrated SCLC

**FIGURE 2:**
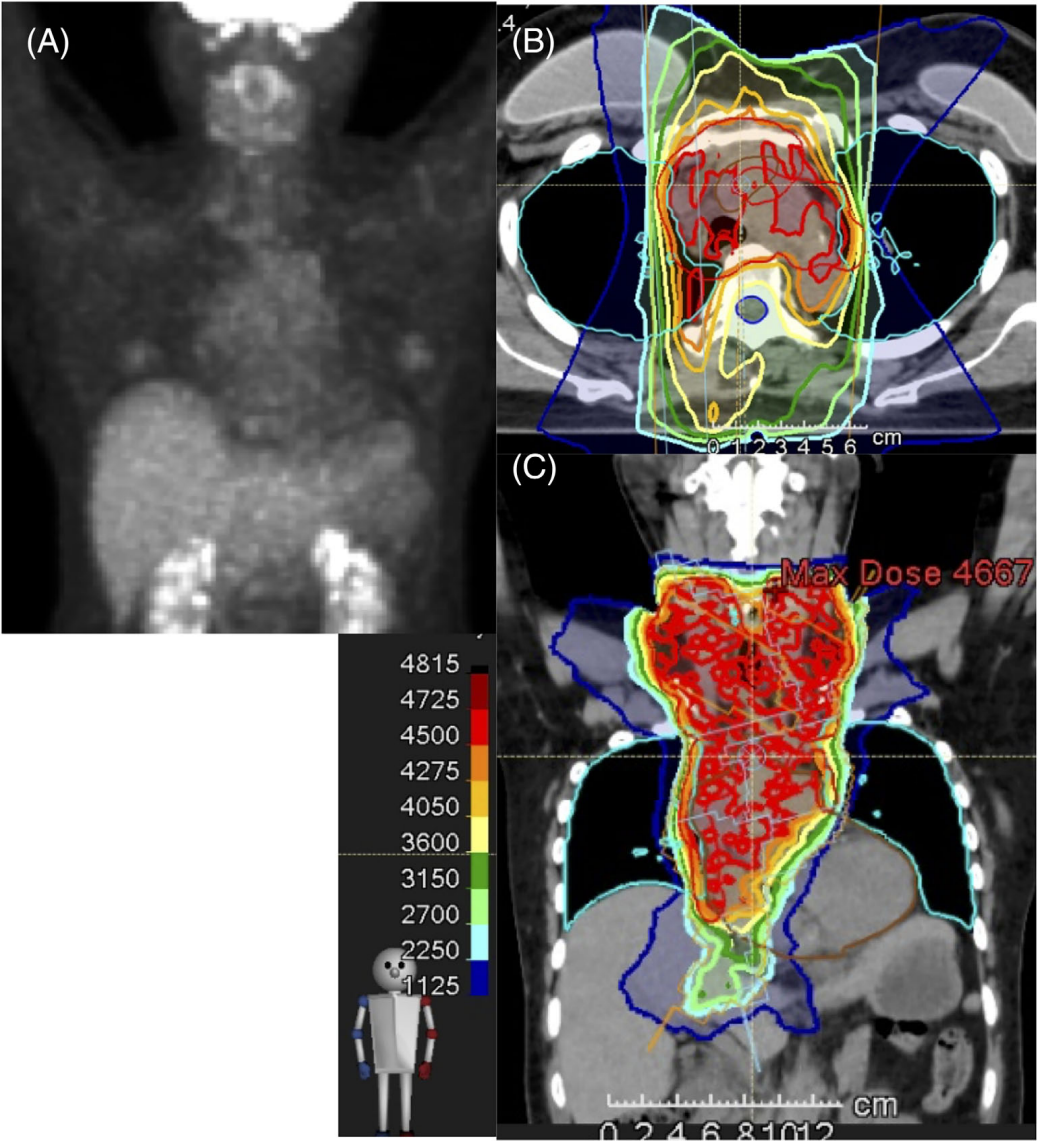
Positron emission tomography‐computed tomography (PET‐CT) images after two cycles of chemotherapy and dose distribution images of the volumetric modulated arc therapy (VMAT) plan. (A) Anterior view of the maximum intensity projection (MIP) image of the PET‐CT scan revealed little accumulation compared with that of pretreatment. (B, C) Transaxial and coronal dose distribution images and dose colour wash of the VMAT plan. V20, V5 and mean lung dose of the VMAT plan were 26.23%, 63.42% and 1403 cGy, respectively

The patient was a 50‐year‐old woman. She visited the Department of Otolaryngology at a local general hospital for a lump in the neck in February of a certain year. The biopsied specimen of the right cervical LN showed SCLC; positron emission tomography‐computed tomography (PET‐CT) revealed accumulation of the right middle lobe tumour and the bilateral supraclavicular, bilateral mediastinal and right pericardiac LNs with very small amount of right pleural effusion (Figure 1); and brain magnetic resonance imaging (MRI) showed no metastasis. The patient with ES‐SCLC, cT1cN3M1b (LYM), was referred to our hospital in the same month. She had a high tumour marker level of Neuron‐specific enolase (NSE) 183 ng/ml (normal range, <16.3 ng/ml) and aspiration cytology of the left supraclavicular LN revealed SCLC (Figure 1). Three days later, chemotherapy with CDDP plus etoposide was started, and PET scan for re‐evaluation after two cycles of chemotherapy showed little accumulation compared with that of pretreatment (Figure 2). Forty‐five Gy of volumetric modulated arc therapy, which is an extension method to dynamic multi‐leaf collimator IMRT at 1.5 Gy twice a day, was added to the third cycle of chemotherapy (Figure 2). V20 (volume of lung receiving 20 Gy or more), V5 (volume of lung receiving 5 Gy or more) and a mean lung dose are 26.23%, 63.42% and 1403 cGy, respectively. The fourth cycle of chemotherapy had to be delayed due to G2 radiation oesophagitis and G4 neutropenia. Since she did not receive prophylactic cranial irradiation (PCI), surveillance with CT and head MRI was performed every 3–6 months for the first 2 years of treatment, and CT scan at 2 years and 3 months after the last chemotherapy confirmed that the complete response had been maintained. The tumour marker NSE has been stable within normal limits for more than 2.5 years.

## DISCUSSION

The seventh and the eighth editions of the TNM staging system are useful in the treatment of SCLC.^3^, ^4^ Stages I–III and stage IV in these systems correspond to LS and ES in the VALSG staging system. In the seventh edition, intrathoracic and extrathoracic metastases were classified as M1a and M1b, respectively. M1b in the seventh edition was divided into M1b and M1c in the eighth edition. In the eighth edition, M1b was defined as involvement of a single extrathoracic metastasis, while newly created M1c was defined as involvement of multiple extrathoracic metastases. Lung cancer that extends to an intrathoracic nonregional LN beyond the nodal stations of the IASLC LN chart is considered as a distant metastasis (M1b disease).^4^ M1b disease had a similar prognosis to intrathoracic metastases (M1a disease), and a better prognosis than M1c disease. Single‐site metastases (SSM) to the brain alone had a better prognosis than SSM to other sites,^4^ but the prognosis for patients with intrathoracic nonregional LN metastases, such as our case, was unclear due to rarity.

Consolidative TRT is recommended for ES‐SCLC patients who had a complete or good response to chemotherapy.^5^, ^6^ While platinum‐based chemotherapy is the mainstay of treatment for ES‐SCLC, the CREST phase III trial was conducted to evaluate TRT (30 Gy in 10 fractions) in ES‐SCLC patients who responded to chemotherapy. There was a significant reduction in intrathoracic recurrence and improved 2‐year survival in the intervention group compared to the control group. A post hoc analysis showed patients with two or fewer distant metastases had better survival after TRT, suggesting that this approach should be considered in ES‐SCLC patients with residual thoracic disease and low‐volume extrathoracic metastases who have a complete or good response to chemotherapy. On the other hand, in the recent NRG Oncology RTOG 0937 phase II trial, patients with ES‐SCLC and one to four extracranial metastases after a complete or partial response to chemotherapy were randomized to PCI alone or PCI plus TRT (45 Gy in 15 fractions) for intrathoracic disease and/or consolidative radiation (30–45 Gy) for extracranial metastases. Although this trial was terminated at interim analysis because of slow recruitment and no significant differences in 1‐year overall survival (OS), the lower risk of first thoracic recurrence and higher proportion of patients with failures at any new sites in the TRT group suggested the need for better systemic therapy and RT including timing, dose and fractionation of RT. In this regard, the CALGB 30610 trial, which compared dose escalating TRT of 70 Gy in 35 fractionations to accelerated hyperfractionated (AHF)‐TRT of 45 Gy twice daily in 30 fractions (the Turrisi method), showed no significant difference in OS and progression‐free survival (PFS),^8^ supporting high‐dose once‐daily RT as an acceptable option for patients with LS‐SCLC. This notion is also supported by the CONVERT study. Furthermore, in a Norwegian phase 2 study,^9^ the experimental arm involving high‐dose (60 Gy in 40 fractions) AHF‐TRT resulted in a substantial survival improvement without increased toxicity, compared with 45 Gy of the Turrisi method, suggesting that AHF‐TRT with 60 Gy is an alternative to the Turrisi method in LS‐SCLC.

The number of metastases is a prognostic factor, and the majority of the long‐term survivors with ES‐SCLC had been reported to have either a single metastatic site or metastases limited to contralateral hemithorax and/or contralateral cervical or axillary nodes.^10^ In addition, subsequent subgroup analyses of the CREST trial revealed that the OS benefit of TRT was limited to patients with residual thoracic disease, and the PFS of TRT was conferred to patients with two or fewer metastatic sites and no liver or bone metastases. Furthermore, the retrospective study, using a large cohort of patients from the National Cancer Database, showed a significant difference in survival with the additional use of TRT with chemotherapy and also with greater number of radiation treatments (higher radiotherapy doses).^11^ As our case was controlled by IMRT and chemotherapy, ED‐SCLC patients with SSM such as contralateral cervical or axillary or intrathoracic nonregional LN metastasis, excluding liver or bone metastases, might be good candidates for chemo‐IMRT. As IMRT is performed from multiple directions, the area of low‐dose irradiation increases, and also attention should be paid to radiation pneumonitis and the risk of secondary cancer. The combination of chemotherapy and consolidative TRT while managing systemic toxicities for ES‐SCLC patients might be the paradigmatic model of multidisciplinary treatment.

For the first‐line treatment of ES‐SCLC, chemoimmunotherapy of platinum and etoposide combined with anti‐PD‐L1 antibodies including atezolizumab and durvalumab has been shown to improve survival and has become the standard of care.^1^, ^5^, ^6^, ^7^ In this treatment setting, the role of consolidative TRT is less clear. In this regard, a phase II/III RAPTOR trial (NRG Oncology LU‐007) is very important which compares the effect of adding RT (up to five sites including primary thoracic disease) to the usual maintenance therapy with atezolizumab versus atezolizumab alone in ES‐SCLC patients without progressive disease after four to six cycles of platinum plus etoposide chemotherapy combined with atezolizumab.

In SCLC, better local and systemic therapies are necessary to improve OS. We here present a case of a female ES‐SCLC patient with right pericardial LN involvement. To our knowledge, this is the first case report of intrathoracic ES‐SCLC, who is successfully treated with IMRT and concurrent chemotherapy. Continued advances in multimodal therapy are essential for the optimal clinical management of ES‐SCLC.

## CONFLICT OF INTEREST

None declared.

## AUTHOR CONTRIBUTION

All authors contributed to the patient's therapy and/or manuscript preparation. Waki Hosoda also participated in the histological diagnosis. All the authors have read the manuscript and have approved this submission.

## ETHICS STATEMENT

The authors declare that appropriate written informed consent was obtained for the publication of this manuscript and accompanying images.

## Data Availability

The data that support the findings of this study are available on request from the corresponding author. The data are not publicly available due to privacy or ethical restrictions.
